# Prediction models for progression from prediabetes to diabetes: a systematic review and meta-analysis

**DOI:** 10.3389/fendo.2026.1888466

**Published:** 2026-07-08

**Authors:** Yanxian Wang, Cuili Wang, Jianxiu Wang

**Affiliations:** 1 School of Preschool Education, Qingdao Preschool Education College, Qingdao, China; 2 Department of Emergency Medicine, Qilu Hospital (Qingdao), Cheeloo College of Medicine, Shandong University, Qingdao, China

**Keywords:** machine learning, meta-analysis, prediabetes, prediction model, systematic review, type 2 diabetes mellitus

## Abstract

**Purpose:**

Prediabetes increases the risk of type 2 diabetes mellitus (T2DM). Accurate prediction is crucial for early prevention, but evidence on prediction models has not been comprehensively synthesized. This study systematically evaluated the accuracy of such models in predicting prediabetes-to-T2DM progression.

**Methods:**

Databases including Cochrane Library, Embase, PubMed, and Web of Science were searched up to June 2, 2025. PROBAST was applied to evaluate the risk of bias. STATA 15.0 was employed to analyze the pooled concordance index (C-index) with 95% CI, to conduct subgroup and sensitivity analyses, and to assess publication bias.

**Results:**

Sixteen studies were included, covering 1,368,130 prediabetic individuals with 187,225 progressing to T2DM. Pooled incidence was 42.3‰ (95% CI: 27.2‰–60.4‰). Pooled C-indices of the training and validation sets were 0.76 (0.71–0.80) and 0.84 (0.82–0.86), respectively. Logistic regression and random forest yielded C-indices of 0.81 and 0.86, respectively.

**Conclusions:**

Prediction models show promising accuracy for predicting progression from prediabetes to T2DM, although the evidence remains limited, particularly due to the lack of external validation. Future research should strengthen model development, external validation, and reporting quality to improve the robustness and clinical applicability of prediction models for the progression of prediabetes.

**Systematic Review Registration:**

https://www.crd.york.ac.uk/PROSPERO/, identifier CRD420251104222.

## 1 Introduction

Type 2 diabetes mellitus (T2DM) constitutes a major public health concern worldwide. It is marked by its prevalence as a chronic metabolic disorder (1, 2). According to recent estimates from the Global Burden of Disease 2021 Diabetes Collaborators, the age-standardized global prevalence of diabetes mellitus (DM) was 6.1% in 2021. Of those cases, 96.0% were T2DM. Projections suggest the number of individuals with T2DM will exceed 1.27 billion by 2050, and the age-standardized prevalence will rise to 9.5% (3). The burden of this disease is not uniformly distributed, exhibiting significant geographical disparities. Regions with relatively scarce healthcare resources, particularly rural areas, often have higher prevalence rates and associated mortality (4, 5). Chronic hyperglycemia drives the development of severe microvascular and macrovascular complications, including retinopathy (6), nephropathy (7), neuropathy (8), and cardiovascular disease (9). These complications substantially diminish life quality and place significant economic strain on healthcare infrastructures (10). Consequently, effective early prevention and intervention for T2DM has profound clinical and societal importance.

The pathogenesis of T2DM is multifactorial, influenced by factors such as age, obesity, and physical inactivity. Among these, the prediabetic state—an intermediate metabolic condition where blood glucose levels exceed the normal range but fall below the diagnostic threshold for DM (11, 12)—constitutes a critical, independent risk factor for progression to T2DM (13). Pathologically, this state involves concurrent insulin resistance and β-cell dysfunction (14). Individuals with prediabetes face an approximate 10% annual risk of progressing to T2DM. They also face a markedly elevated risk for cardiovascular disease and all-cause mortality (15–18). While lifestyle interventions have demonstrated efficacy in DM prevention, a persistent challenge remains. That is to accurately identify those individuals at the highest progression risk within the vast prediabetic population to enable efficient, targeted intervention. Effective risk prediction tools are therefore essential to facilitate personalized risk stratification, optimize resource allocation, and advance precision prevention strategies.

Advances in artificial intelligence, particularly machine learning (ML), offer considerable potential in this field. ML algorithms excel at handling high-dimensional, multi-source data—such as electronic health records, genomics, metabolic profiles, and lifestyle data—to identify complex, non-linear relationships and construct accurate predictive models (19–21). Existing research has applied various algorithms, including support vector machines (SVM), random forest (RF), and neural networks. Favorable discriminative performance is observed in such studies (22–24). Meanwhile, traditional statistical prediction models, including logistic regression (LR), Cox proportional hazards regression, and nomogram-based models, have also been extensively investigated and remain important tools for risk prediction because of their interpretability, transparency, and widespread clinical applicability. These models typically utilize variables encompassing demographic characteristics, metabolic indices, lifestyle factors, and genetic data to provide quantitative risk assessment. Recent efforts have begun applying ML specifically to predict progression from prediabetes to T2DM (25, 26). However, a systematic synthesis summarizing the current application and effectiveness of prediction models for this specific transition is currently lacking. This poses a challenge for formulating targeted screening strategies. Our meta-analysis is therefore conducted to extensively evaluate the value of such models in predicting progression from prediabetes to T2DM. The objective is to provide a scientific foundation for developing personalized and generalizable early-warning tools.

## 2 Methods

### 2.1 Study registration

This systematic review and meta-analysis was conducted and reported in accordance with the Preferred Reporting Items for Systematic Reviews and Meta-Analyses (PRISMA) 2020 Statement (27). The research protocol was prospectively registered on the International Prospective Register of Systematic Reviews database (CRD: 420251104222) to ensure transparency and reproducibility. A complete PRISMA 2020 checklist is provided in the **Supplementary Materials**.

### 2.2 Eligibility criteria

#### 2.2.1 Inclusion criteria

- Population: Study populations with a clear diagnosis of prediabetes based on recognized guidelines (e.g., American Diabetes Association, World Health Organization).
- Intervention: Research constructing prediction models to predict progression from prediabetes to T2DM.
- Control: The control group comprised individuals with prediabetes who did not develop T2DM during the follow-up period.
- Outcomes: Studies reporting discrimination metrics for model evaluation, such as the concordance index (C-index), area under the receiver operating characteristic curve (AUC), sensitivity, specificity, confusion matrix, precision, or accuracy.
- Time: No restriction was placed on the follow-up duration of the included investigations.
- Study design: Observational studies were eligible, encompassing cohort studies, case-control studies, and cross-sectional studies. Only studies published in English were considered.

#### 2.2.2 Exclusion criteria

- Research failing to strictly distinguish prediabetic from other populations.
- Studies conducting only risk factor analysis without constructing a complete predictive model.
- Conference abstracts published without formal peer review.
- Validation studies of established scale-based tools.

### 2.3 Data sources and search strategy

A systematic retrieval was conducted across the Cochrane Library, Embase, PubMed, and Web of Science up to June 2, 2025. The retrieval used both Medical Subject Headings and free-text keywords, with no restrictions on region or publication date. Reference lists of relevant review articles were also screened. Specifics are provided in **Supplementary Table 1**.

### 2.4 Study selection

Records identified from the database search were imported into EndNote 21, and duplicates were removed. Two independent reviewers (Wang YX and Wang JX) carried out a title/abstract screening for the remaining articles based on the pre-defined eligibility criteria. Potentially eligible studies underwent a full-text review for final eligibility. Discrepancies were settled by discussion with a third reviewer.

### 2.5 Data extraction

Two independent reviewers (Wang YX and Wang JX) extracted data from the eligible studies. Discrepancies were addressed by consensus involving a third reviewer. Extracted information comprised: first author, DOI, title, study design, publication year, follow-up duration, country, patient source, prediabetes diagnostic criteria, predicted event, number of events, total sample size, training set event and sample size, validation set generation method, overfitting prevention methods, validation set event and sample size, missing data handling, feature selection method, model types, and modeling variables.

### 2.6 Risk of bias (RoB) in studies

Using the Prediction Model Risk Of Bias Assessment Tool (PROBAST), the RoB of eligible studies was evaluated. This tool comprises a series of questions organized into four distinct domains: participants, predictors, outcomes, and statistical analysis, collectively reflecting the overall RoB and applicability. The four domains encompass two, three, six, and nine specific questions, respectively. Each question is answered using one of three options: “yes/probably yes,” “no/probably no,” or “no information.” A domain is classified as being at high risk if it contains at least one question answered “no” or “probably no.” To be rated as low risk, a domain must have all questions answered “yes” or “probably yes.” The overall RoB is judged as low only when all domains are considered low risk. Conversely, the overall assessment is high risk if at least one domain is rated as high risk.

Two independent investigators performed the bias risk assessment using PROBAST. They then cross-checked their evaluations. Discrepancies were settled by consultation with a third investigator.

### 2.7 Synthesis methods

Meta-analysis was performed on the C-index, the primary metric for evaluating overall model discrimination. For studies reporting a C-index without its 95% confidence interval (CI) or standard error, the method described by Debray et al. was applied for estimation (28). Heterogeneity was quantified using the I² statistic. A random-effects model was used if I² > 50%; otherwise, a fixed-effects model was applied. Publication bias was assessed utilizing funnel plots and Egger’s test. For the meta-analysis of incidence rates, a double arcsine transformation was applied prior to pooling. To address the dependence introduced by multiple effect sizes from a single study, a sensitivity analysis was performed by retaining only one model per study. Priority was given to the metrics of model performance from the largest sample or the external validation set; when neither was available, the optimal model recommended by the authors or the one exhibiting the highest C-index was selected. The C-index was then re-aggregated based on a single effect size per study and compared with the primary analysis. A P < 0.05 indicated statistical significance.

## 3 Results

### 3.1 Study selection

**Figure 1:**
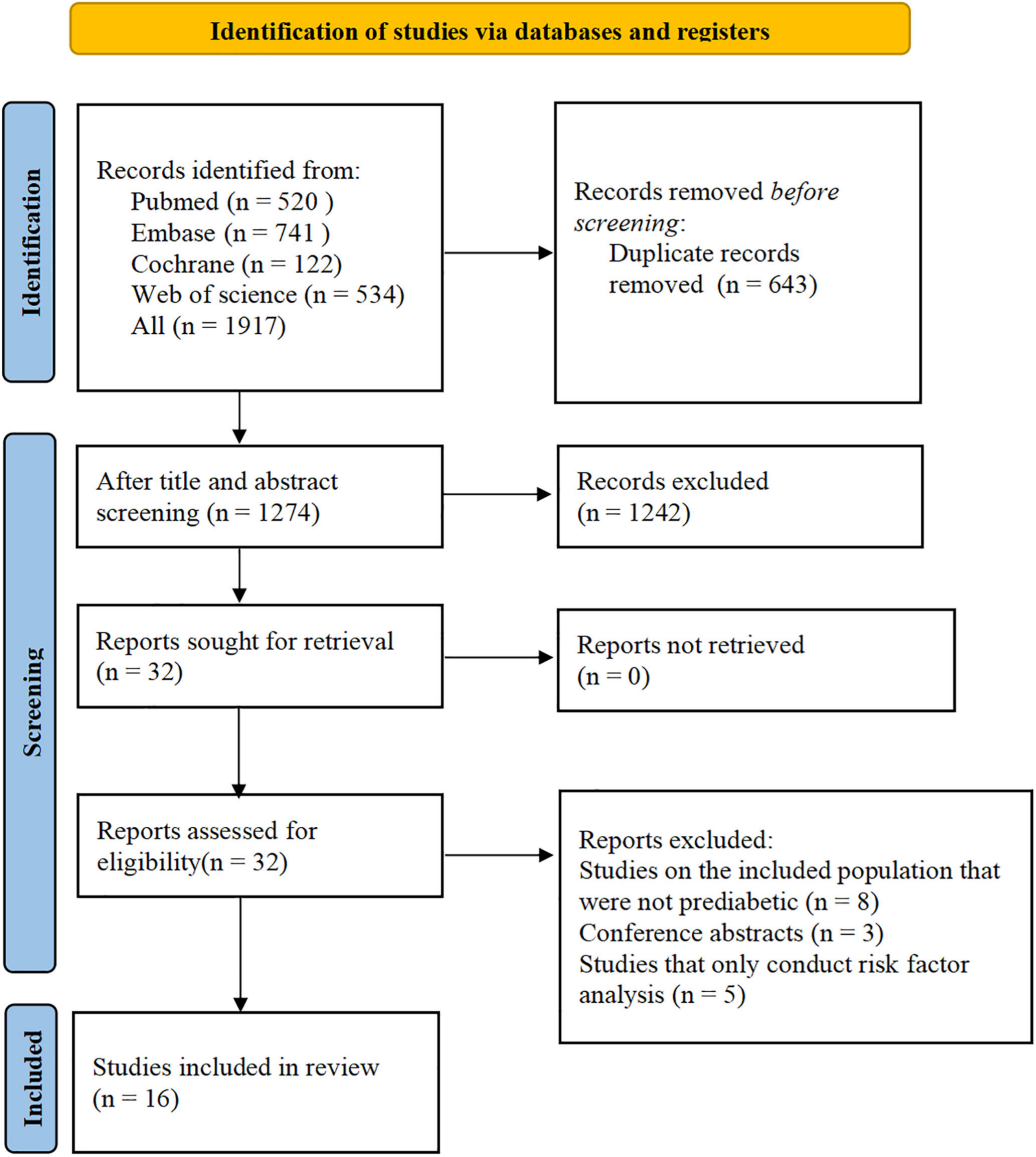
PRISMA flow diagram of literature screening.

The database retrieval produced 1,917 articles. Following the removal of 643 duplicates, the titles and abstracts of 1,274 records were screened. Of these, 1,242 irrelevant records were eliminated. The remaining 32 articles were reviewed in full text. Eight studies were removed for not focusing on a prediabetic population, three for being non-peer-reviewed conference abstracts, and five for only performing risk factor analysis. Finally, 16 articles were included. Specifics are presented in the PRISMA flow diagram (**Figure 1**).

### 3.2 Study characteristics

The 16 eligible studies involved 1,368,130 individuals with prediabetes, with sample sizes ranging from 1,857 to 1,217,792. Among these individuals, 187,225 progressed to T2DM. The studies originated from multiple countries, including the United States (U.S.), Israel, Spain, China, Japan, Denmark, and Switzerland. Publication dates spanned 2019 to 2025. Study designs comprised two cross-sectional and 14 cohort studies. Patient sources were single-center (n = 8), multi-center (n = 3), and registry databases (n = 5). The predicted outcome for all studies was progression to T2DM. All eligible studies specified the diagnostic criteria of prediabetes. Follow-up duration ranged from two to ten years. All 16 studies explicitly described the validation set generation method: random split (n = 6), cross-validation (n = 8), internal validation (n = 1), and external validation (n = 1). A variety of prediction models were evaluated in these studies. Modeling variables were commonly available and interpretable clinical features (**Supplementary Table 2**).

### 3.3 RoB in studies

**Figure 2:**
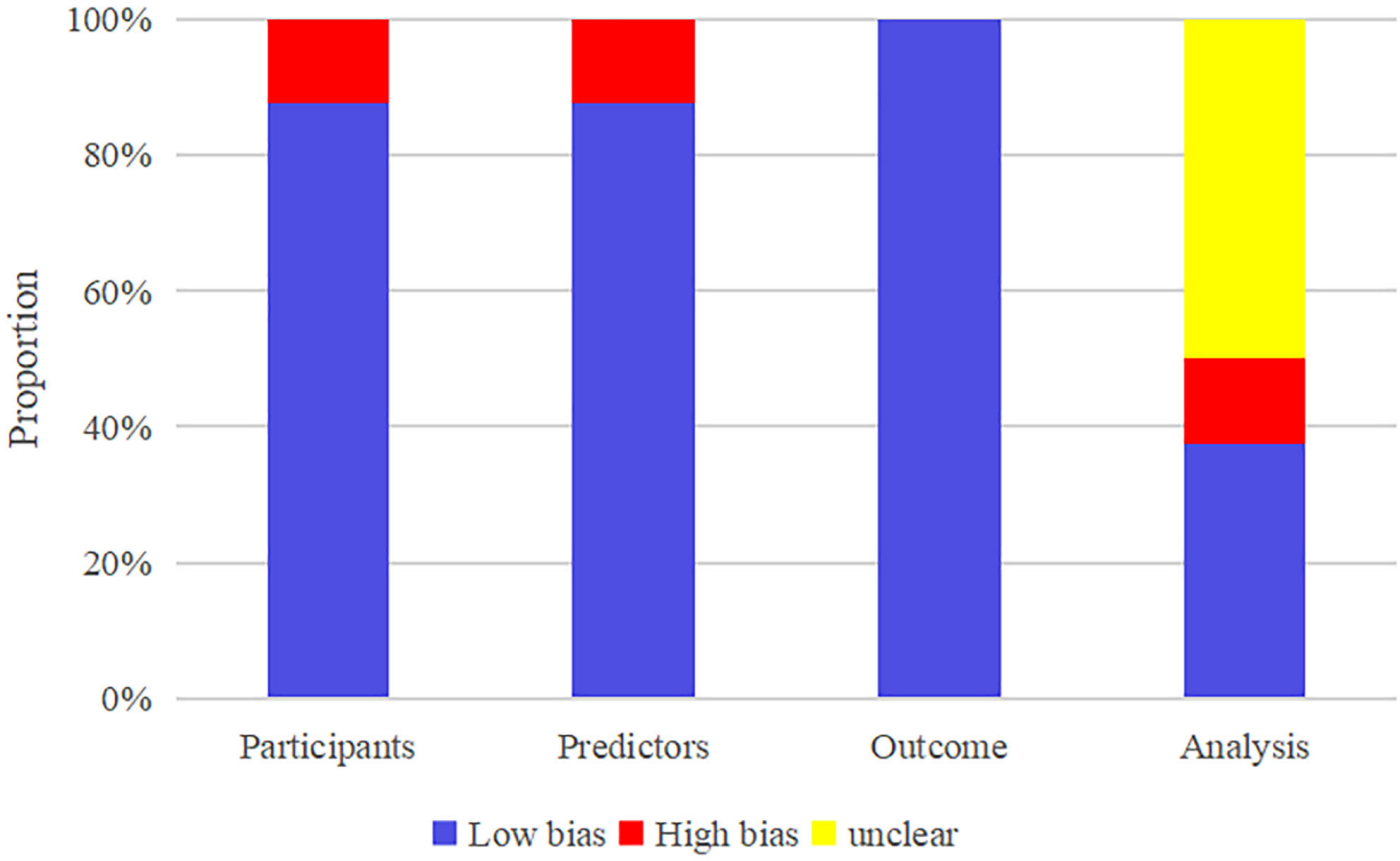
RoB assessment of the eligible studies using PROBAST.

The 16 studies were evaluated for RoB using PROBAST across four domains (participants, predictors, outcome, analysis). In the participants domain, the two cross-sectional studies were rated as high risk due to their design, while the 14 cohort studies were of low risk. In the predictors domain, two studies were rated as high risk due to inconsistent predictor definition/assessment; 14 were of low risk. The outcome domain was rated as low risk for all studies, as prediabetes was defined per accepted guidelines and patients’ glucose status at the end of follow-up was not used. Regarding the analysis domain, two studies were rated as high risk for deleting missing data directly, eight were rated as having unclear risk for not reporting methods to avoid overfitting, and six were low risk due to appropriate sample size, variable handling, and validation methods. In summary, the main sources of bias were concentrated in participant selection, predictor assessment, and statistical analysis procedures. The outcome domain showed minimal bias. However, several studies were judged to have a high or unclear RoB in the analysis domain, primarily due to inadequate reporting of overfitting prevention methods and inappropriate handling of missing data. Therefore, the overall RoB among the included studies remains considerable, and the findings should be interpreted with caution (**Figure 2**).

### 3.4 Meta-analysis of incidence rates

**Figure 3:**
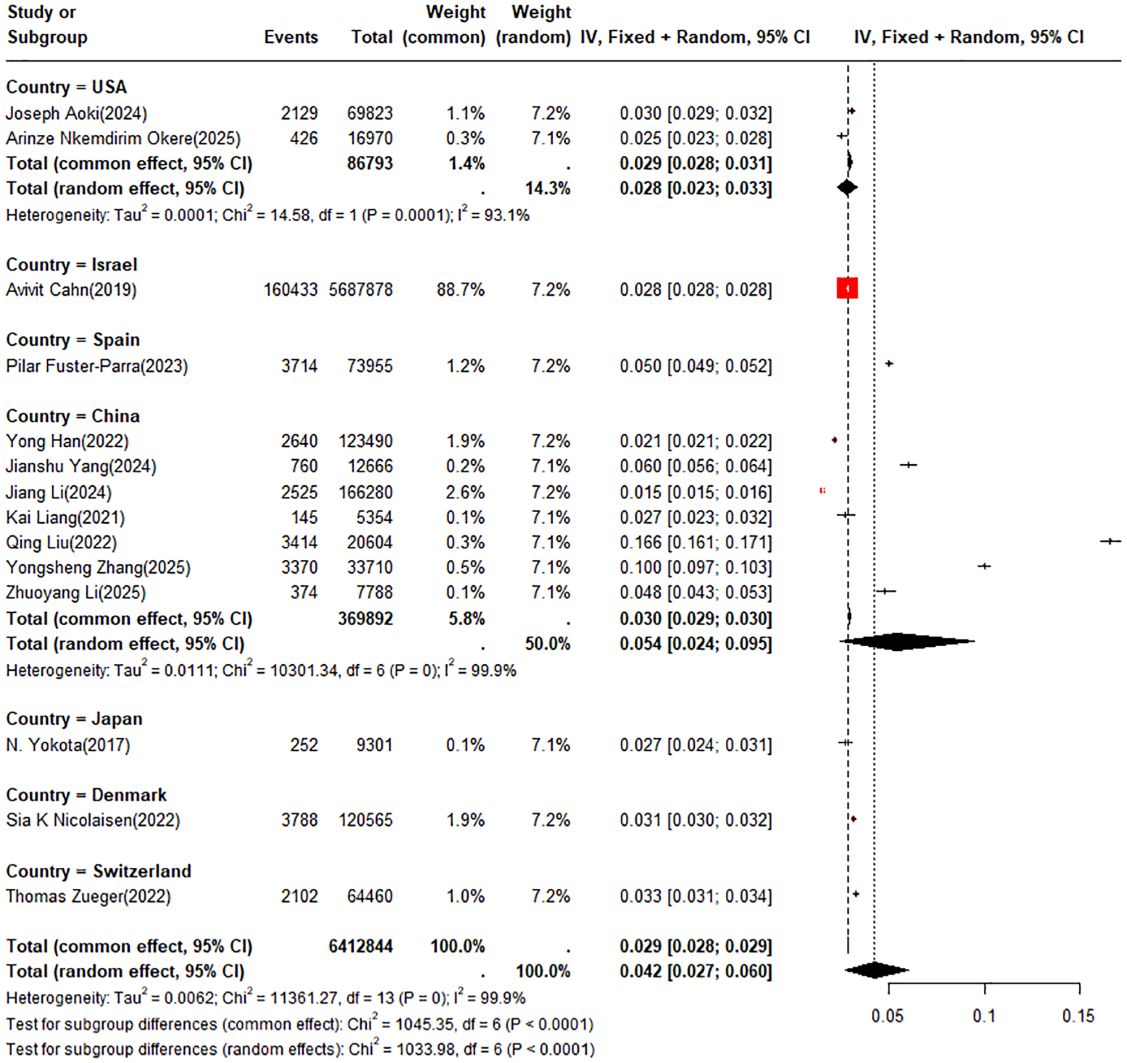
Forest plot of the meta-analysis for the incidence of progression from prediabetes to T2DM.

Fourteen studies reported follow-up duration and the number of T2DM cases during follow-up. The pooled incidence, computed using a random-effects model, was 42.3 per 1000 person-years (95% CI: 27.2‰ to 60.4‰). Stratified by region, the incidence was 54.2 per 1000 person-years (95% CI: 24.4‰ to 95.0‰) in Chinese populations and 27.8 per 1000 person-years (95% CI: 22.8‰ to 33.4‰) in U.S. populations (**Figure 3**).

### 3.5 Meta-analysis of predictive models

**Figure 4:**
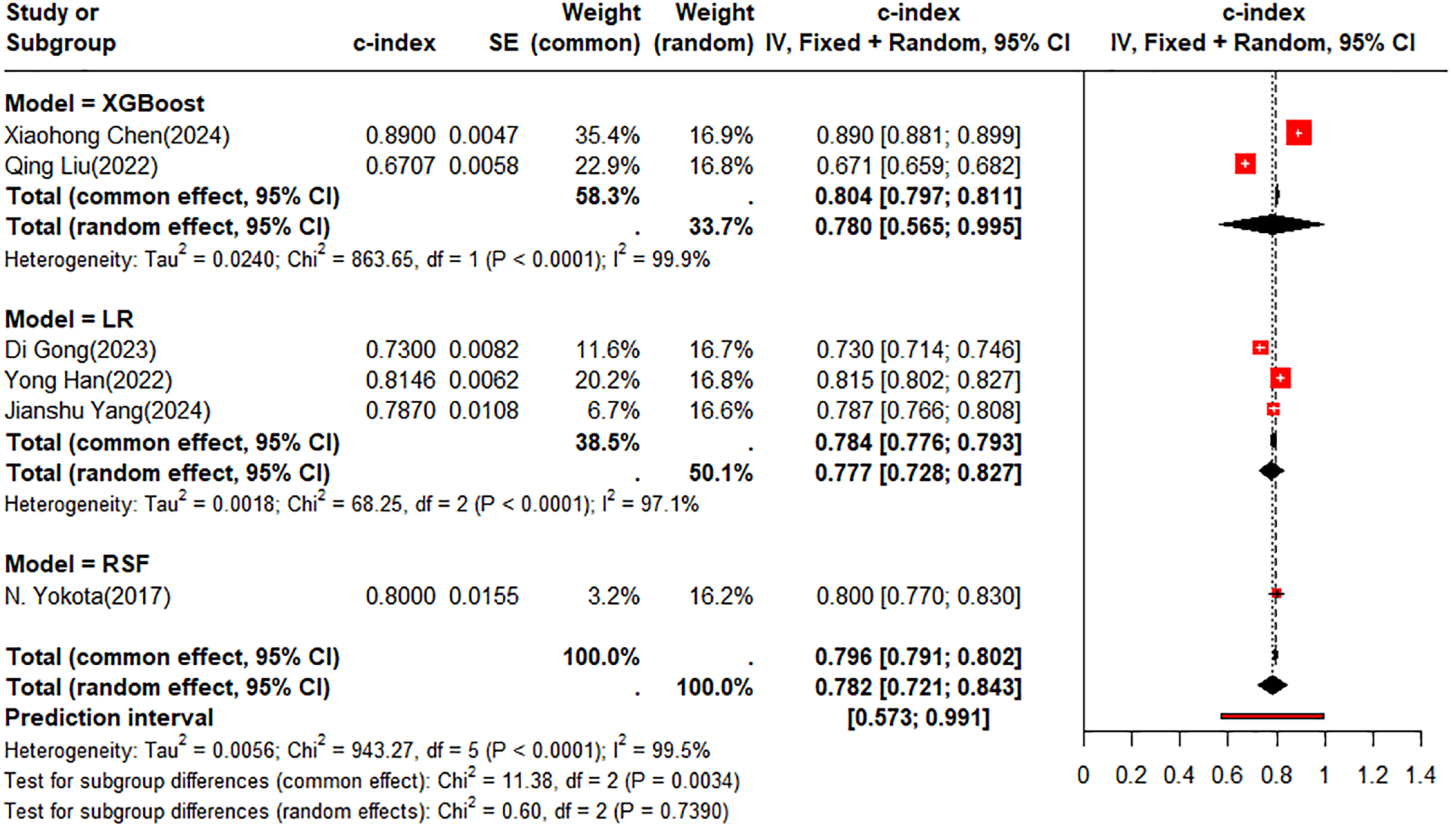
Forest plot of the meta-analysis for the C-index of prediction models in predicting progression from prediabetes to T2DM (training set).

Six studies reported model performance on training sets, covering three model types: LR, random survival forest (RSF), and extreme gradient boosting (XGBoost). The pooled C-index for the training set was 0.782 (95% CI: 0.721–0.843). Subgroup analysis by model type yielded a C-index of 0.777 (95% CI: 0.728–0.827, n = 3) for LR, 0.780 (95% CI: 0.565–0.995, n = 2) for XGBoost, and 0.800 (95% CI: 0.770–0.830, n = 1) for RSF (**Figure 4**). Subgroup analysis by model type yielded a C-index of 0.777 (95% CI: 0.728–0.827, n = 3) for LR, 0.780 (95% CI: 0.565–0.995, n = 2) for XGBoost, and 0.800 (95% CI: 0.770–0.830, n = 1) for RSF (**Figure 4**).

**Figure 5:**
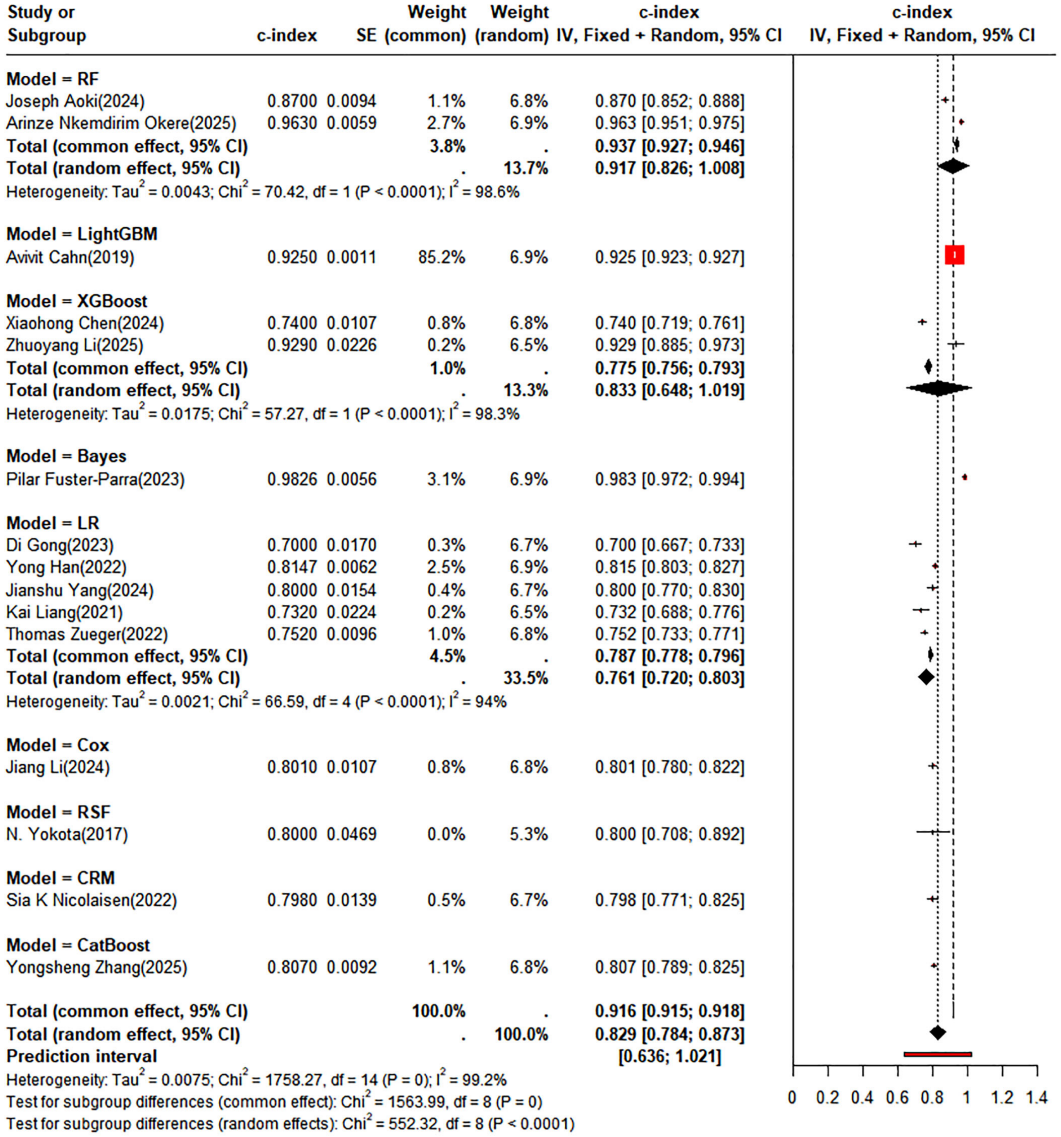
Forest plot of the meta-analysis in the validation set for the C-index of prediction models in predicting progression from prediabetes to T2DM (subgroup analysis of model type).

Fifteen studies reported model performance on validation sets, covering nine model types: LR, XGBoost, RF, RSF, LightGBM, competing risks model (CRM), Cox, CatBoost, and Bayes. The pooled C-index for the validation set was 0.829 (95% CI: 0.784 – 0.873, n = 15). Subgroup analysis by model type yielded a C-index of 0.761 (95% CI: 0.720–0.803, n = 5) for LR, 0.833 (95% CI: 0.648–1.000, n = 2) for XGBoost, 0.917 (95% CI: 0.826–1.000, n = 2) for RF, 0.800 (95% CI: 0.708–0.892, n = 1) for RSF, 0.925 (95% CI: 0.923–0.927, n = 1) for LightGBM, 0.798 (95% CI: 0.771–0.825, n = 1) for CRM, 0.801 (95% CI: 0.780–0.822, n = 1) for Cox, 0.807(95% CI: 0.789–0.825, n = 1) for CatBoost, and 0.983 (95% CI: 0.972–0.997, n = 1) for Bayes (**Figure 5**; **Table 1**).

**Table 1:** Meta-analysis of the C-index of prediction models in predicting progression from prediabetes to T2DM.

Model	Training set				Validation set			
	n	T2DM cases	Sample size	C-index (95% CI)	n	T2DM cases	Sample size	C-index (95% CI)
Bayes					1	142	16648	0.983 (0.972 - 0.994)
CatBoost					1	760	2157	0.807 (0.789 - 0.825)
Cox					1	505	2698	0.801 (0.780 - 0.822)
CRM					1	267	5201	0.798 (0.771 - 0.825)
LightGBM					1	17922	381872	0.925 (0.923 - 0.927)
LR	3	3327	18688	0.777 (0.728 - 0.827)	5	2846	20977	0.761 (0.720 - 0.803)
RF					2	753	4534	0.917 (0.826 - 1.000)
RSF	1	227	1895	0.800 (0.770 - 0.830)	1	25	210	0.800 (0.708 - 0.892)
XGBoost	2	2110	3217	0.780 (0.565 - 0.995)	2	916	2240	0.833 (0.648 - 1.000)
Overall	6	8388	33407	0.782 (0.721 - 0.843)	15	24136	436537	0.829 (0.784 - 0.873)

T2DM cases - The number of T2DM cases.

**Figure 6:**
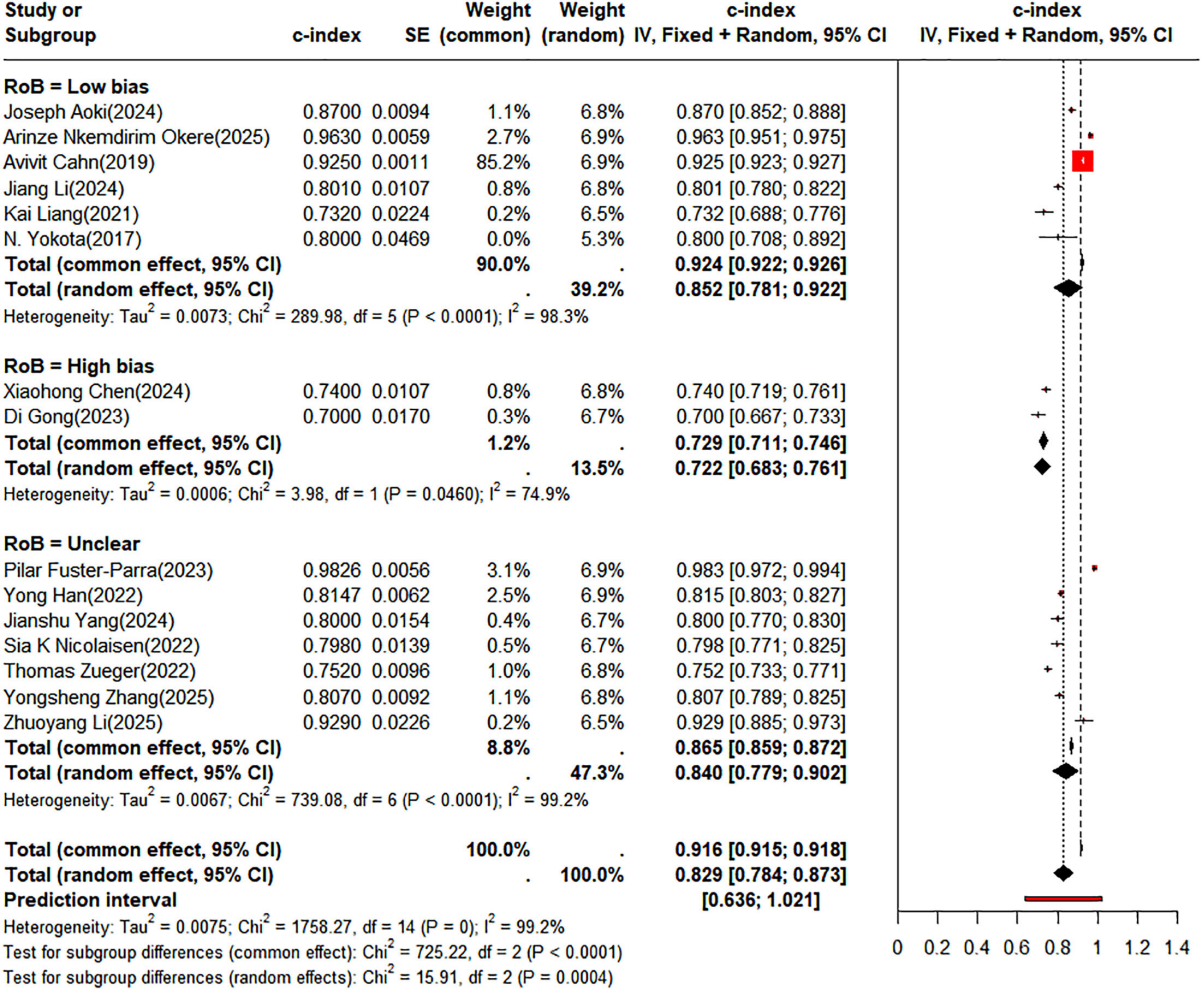
Forest plot of the meta-analysis in the validation set for the C-index of prediction models in predicting progression from prediabetes to T2DM (subgroup analysis of RoB).

Subgroup analysis by RoB revealed that investigations with a low RoB produced a pooled C-index of 0.852 (95% CI: 0.781–0.922, n = 6), those with a high RoB yielded a pooled C-index of 0.722 (95% CI: 0.683–0.761, n = 2), and those with an unclear risk generated a pooled C-index of 0.840 (95% CI: 0.779–0.902, n = 7) (**Figure 6**).

**Figure 7:**
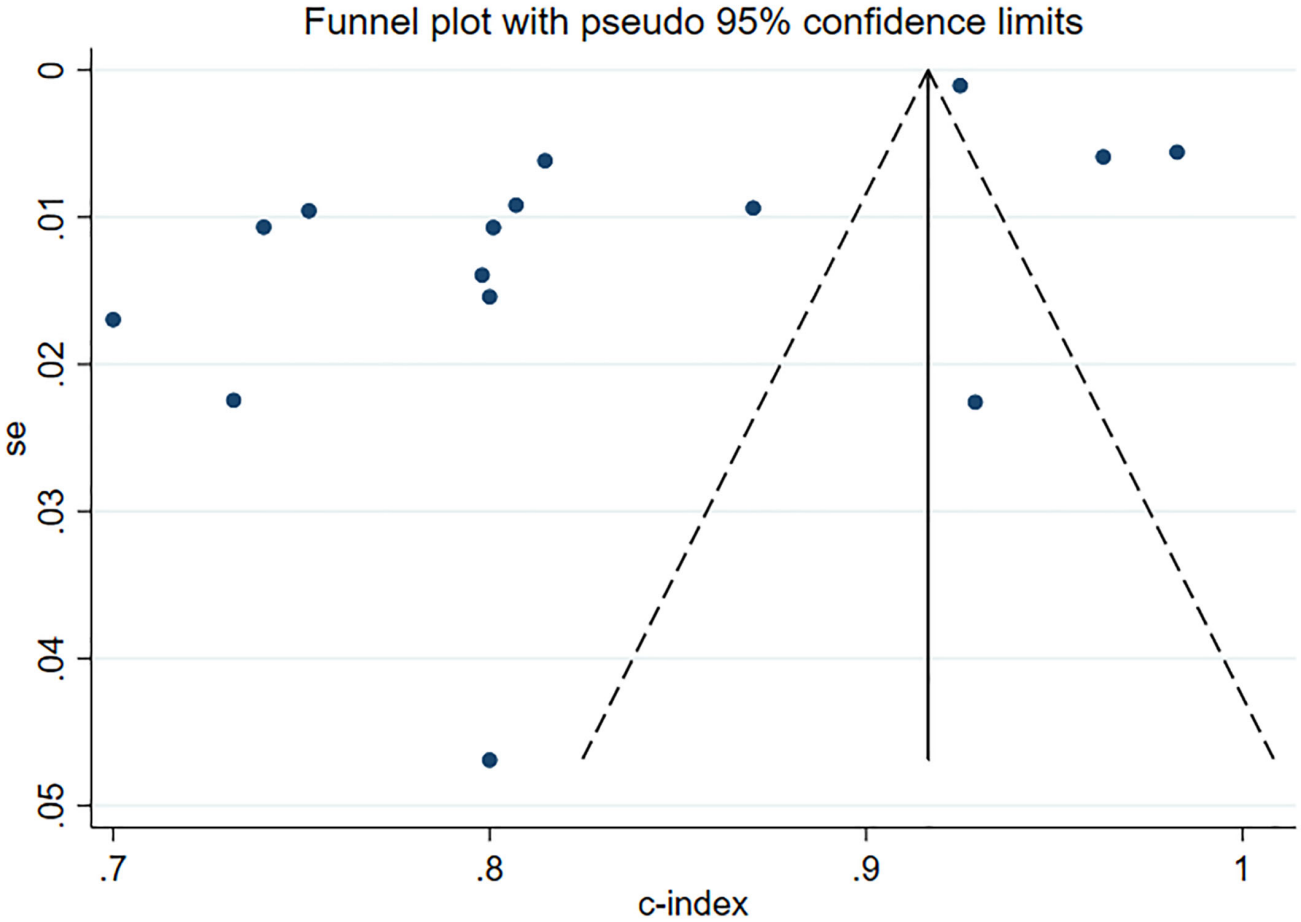
Funnel plot of the original studies for LR in the validation set (subgroup analysis of RoB).

Meta-regression was conducted with study design, the proportion of T2DM cases in the total sample, country, follow-up duration, and diagnostic strategy as covariates. The results indicated that none of these variables accounted for the sources of heterogeneity (**Table 2**). Publication bias was evaluated utilizing funnel plots along with Begg’s and Egger’s tests. The dataset of the LR model was selected for analysis to assess the robustness of the overall results. The symmetry of funnel plots (**Figure 7**) and the significance in Egger’s (P = 0.006) tests indicated no substantial publication bias.

**Table 2:** Meta-regression of the pooled c-index with different covariates (validation set).

Variables	B	se	Z	P	95% CI
Study design					
Cohort (Ref)					
Others	0.030	0.164	0.185	0.854	-0.291 - 0.352
Proportion of T2DM (%)	-0.003	0.003	-0.877	0.380	-0.009 - 0.003
Country					
China (Ref)					
Others	0.042	0.052	0.809	0.419	-0.060 - 0.144
Follow Up (m)	0.005	0.008	0.585	0.559	-0.011 - 0.020
Diagnostic_criteria					
Criteria1 (Ref)					
Others	-0.019	0.059	-0.329	0.742	-0.135 - 0.097

(1) Proportion of T2DM (%) - The proportion of T2DM cases in the total number of cases in the validation set. (2) Criteria1 - Glycosylated hemoglobin 5.7% - 6.4%.

### 3.6 Predictor analysis

All 16 eligible studies constructed prediction models using common, interpretable clinical features. Age and body mass index (BMI) were the most frequently used predictors, each appearing in 14 studies. Other core predictors included sex (13 studies), fasting plasma glucose (FPG, 11 studies), triglycerides (nine studies), serum glucose (eight studies), systolic blood pressure (eight studies), alanine aminotransferase (ALT) (eight studies), high-density lipoprotein cholesterol (HDL-C) (eight studies), and glycated hemoglobin (HbA1c, seven studies). Metabolic and cardiovascular indicators like diastolic blood pressure, total cholesterol, and low-density lipoprotein cholesterol (each in five studies) were also common, highlighting the pivotal role of glucose and lipid metabolism in cardiovascular health during disease progression. Several studies incorporated variables such as family history of DM (four studies), education level (three studies), socioeconomic status (three studies), and waist circumference (three studies), thereby enriching the predictive feature space. This comprehensive predictor set spanned demographic, basic metabolic, liver and kidney function, lipid and glucose profiles, genetic background, and socio-behavioral factors, providing a structured informational foundation for prediction models to identify high-risk individuals from multiple dimensions.

## 4 Discussion

### 4.1 Summary of the main findings

The present meta-analysis synthesized data from 16 studies, encompassing 1,368,130 individuals with prediabetes, among whom 187,225 progressed to T2DM. The pooled incidence of progression was 42.3 per 1000 person-years (95% CI: 27.2‰ to 60.4‰). A notable regional variation was identified: 54.2 per 1000 person-years (24.4‰–95.0‰) in Chinese populations and 27.8 per 1000 person-years (22.8‰–33.4‰) in the U.S. populations. This variation likely stems from differences in population characteristics, follow-up duration, diagnostic criteria, and regional factors. Regarding model performance, various algorithms of prediction models in the validation sets demonstrated promising predictive capability. Pooled C-indices for widely used models were 0.81 (95% CI: 0.77–0.85, n = 14) for LR, 0.86 (0.80–0.93, n = 7) for RF, 0.84 (0.76–0.93, n = 5) for XGBoost, and 0.91 (0.83–0.99, n = 4) for Bayesian models. These results indicate that current prediction models possess a high discriminative accuracy for early risk prediction in this context. Furthermore, the absence of significant publication bias strengthens the reliability of these findings.

### 4.2 Comparison with previous reviews

Prior research supports the potential of prediction models in predicting the risk of T2DM. A systematic review by Zhao et al. (29) reports a pooled C-statistic of 0.82 (95% CI: 0.79–0.86) for prediction models in predicting T2DM after gestational DM. Similarly, Cuthbertson et al. (30) find that the fatty liver index, a traditional score, effectively predicts the risk of DM in overweight and obese populations. A high fatty liver index can increase the risk of T2DM by nearly tenfold. These findings collectively suggest the feasibility and value of prediction models. The present analysis builds upon this foundation by specifically focusing on the prediabetic population, providing a novel perspective for early identification and intervention of T2DM.

### 4.3 Predictor relevance

All studies included in this systematic review employed commonly available and interpretable clinical features for constructing prediction models. The analysis revealed that age and BMI were the most widely utilized predictors, each incorporated in 14 studies. This highlights the central role of basic demographic and metabolic indicators in forecasting disease progression. Other metabolic parameters, including sex, FPG, triglycerides, serum glucose, systolic blood pressure, ALT, and HDL-C, were also extensively adopted. Together with age and BMI, these metabolic indicators formed the core feature set of the prediction models. These findings are strongly aligned with established biological evidence from prior research. A Danish study identified that among individuals with obesity and prediabetes, a BMI ≥ 30 kg/m² was associated with higher HbA1c levels (31). Research involving 5,741 participants aged over 40 demonstrated a positive correlation between increasing age and DM progression (32). Multiple observational studies have confirmed that elevated risks of disease progression in prediabetic patients are significantly linked to higher BMI (33), increased levels of FPG and HbA1c (34, 35), reduced HDL-C, and elevated triglycerides (34, 36). Abnormal liver function (e.g., elevated ALT) and increased blood pressure further accelerate DM development by exacerbating insulin resistance and metabolic dysregulation (34, 36). However, current models still insufficiently incorporate novel biomarkers, genetic information, and environmental exposures. Future research should integrate multi-omics data and dynamic monitoring information to develop risk assessment systems with enhanced predictive accuracy and clinical applicability.

### 4.4 Model types

The prediction models included in this systematic review were broadly classified into two categories: survival analysis models and non-survival analysis models. Survival analysis models, including Cox and RSF, are appropriate for handling time-to-event data and are better suited for assessing the dynamic progression risk of prediabetic patients during follow-up (37). Non-survival analysis models, encompassing LR, DT, RF, SVM, XGBoost, LightGBM, CatBoost, ANN, and various Bayesian models, are commonly applied to predict the risk of T2DM at specific time points, such as three or five years (38). Among these, LR remains the most widely used baseline model due to its simple structure and strong parameter interpretability (39). Ensemble learning algorithms, such as RF, XGBoost, and LightGBM, have demonstrated superior discriminative performance in multi-center, large-sample studies. However, complex prediction models, including ANN and ensemble tree models, pose a significant challenge. Despite the potential advantage of these models in predictive accuracy, their inherent “black-box” nature limits clinical interpretability and practical adoption (40). Although some studies have employed techniques like SHapley Additive exPlanations analysis to enhance model interpretability, most original investigations still lack in-depth clarification of variable effect directions, non-linear relationships, and clinically meaningful thresholds. This gap presents a substantial barrier to the broader implementation and generalizability of these models in real-world clinical settings (34).

In the validation set analysis, the conventional LR model exhibited satisfactory discriminative performance (pooled C-index = 0.81) and was not markedly surpassed by more complex ML models such as RF and XGBoost. Statistical inference models like LR possess inherent interpretability and transparency. This makes their predictions easier for clinicians to comprehend, trust, and deploy, given the lower implementation threshold. Moreover, the modeling variables of the included models were all derived from common, interpretable clinical features (e.g., age, BMI, FPG, and blood lipids). As these features are routinely measured in clinical practice across diverse countries and regions, they facilitate cross-population and cross-regional transportability of the model. Nonetheless, a critical shortcoming of the current research is the pervasive neglect of model calibration. Many studies did not adequately report or assess the concordance between predicted and observed probabilities, which severely restricts the appraisal of prediction reliability. Future investigations should move beyond the singular pursuit of discrimination improvement and devote greater attention to model interpretability, calibration, clinical utility, and transparent reporting, thereby strengthening clinical users’ confidence in prediction models.

### 4.5 Current challenges

Despite the promising discrimination of prediction models included in this review, several barriers impede their clinical deployment. First, sample sizes varied considerably across studies. While some investigations enrolled large cohorts, others had limited case numbers, including validation sets containing as few as 25 events. Such constrained samples can undermine model robustness and compromise generalizability. Second, the reporting of model calibration was frequently insufficient. This omission obscures the alignment between predicted probabilities and observed risk, which in turn restricts the clinical utility of these models for personalized risk assessment. A further issue pertains to model development. Systematic comparisons of performance across alternative feature sets were seldom conducted, and consistent strategies for hyperparameter tuning were generally not applied. Consequently, it remains uncertain whether the presented models constitute an optimal solution. Finally, the validation paradigm has been predominantly confined to single-center, internal approaches. Although informative for local applicability, this focus provides little evidence regarding performance across varied populations and healthcare settings, due to the absence of multi-center, prospective external validation. To advance the field, future efforts must prioritize expanded sample sizes, rigorous calibration evaluation, refined feature engineering and model development processes, and collaborative external validation studies. These steps are essential for translating predictive models into routine clinical practice.

### 4.6 Advantages and limitations

This meta-analysis is the first attempt to compile evidence on prediction models for forecasting the transition from prediabetes to T2DM. Unlike earlier reviews, which relied on qualitative analysis or isolated metrics, our synthesis provides a quantitative basis essential for constructing precise early-warning systems. Comparing the performance of multiple algorithms in training and validation contexts clarifies the distinct strengths and optimal application scenarios of different model architectures, thereby informing practical selection decisions. Several limitations warrant acknowledgment. First, despite a systematic and comprehensive search strategy, the final set of eligible studies remained limited in number, and most were single-center retrospective investigations. This may restrict the representativeness of the study population, consequently affecting the external generalizability of the findings. Additionally, substantial methodological and clinical heterogeneity existed across the included studies concerning population characteristics, predictor selection, model construction strategies, validation methods, prediction time windows, and follow-up durations. Certain discrepancies in the diagnostic criteria for prediabetes and T2DM were also noted, which may further compromise the comparability of results and contribute to the observed heterogeneity. Second, evidence from external validation of the prediction models is still relatively scarce. Only one investigation undertook external validation, whereas the remainder predominantly relied on approaches of internal validation, such as random splitting and cross-validation. The generalizability and transportability of these models to different regions, healthcare settings, and populations thus await further verification. Moreover, although subgroup analyses were stratified by model type, certain model types (e.g., adaptive boosting, CatBoost, CRM, Extra Trees, and K-nearest neighbors) were represented by only one or two studies. Performance comparisons among different models should therefore be interpreted cautiously, and the corresponding conclusions require corroboration by additional high-quality research. Third, the PROBAST risk-of-bias assessment revealed that some studies had a high or unclear risk within the analysis domain, primarily manifesting as insufficient reporting of overfitting control measures and suboptimal handling of missing data. These methodological deficiencies may inflate the estimated prediction performance, warranting continued caution when interpreting the present results. Fourth, the original studies reported the metrics of model performance incompletely. Most investigations reported only metrics of discrimination (e.g., AUC or C-index), while critical indicators of classification performance, such as sensitivity, specificity, positive predictive value, and negative predictive value, were infrequently documented. Thus, the model performance under varying thresholds of clinical decision cannot be evaluated in depth. Concurrently, the majority of studies did not sufficiently report calibration-related metrics. Therefore, the agreement between predicted and observed risks cannot be systematically assessed. Calibration is a critical determinant of a prediction model’s value in clinical application, and its absence hinders a comprehensive evaluation of overall predictive capacity. Furthermore, evidence concerning clinical utility remained sparse; metrics reflecting practical clinical value, such as decision curve analysis and net benefit, were rarely reported. Consequently, the current investigation could not provide a quantitative synthesis of model calibration and clinical usefulness, and the existing evidence remains insufficient to advocate the direct clinical implementation of the relevant prediction models. Fifth, although funnel plots and publication bias tests did not detect obvious publication bias, the statistical power of these tests was limited due to the small number of studies in several model types; the corresponding results should thus be interpreted with caution. Finally, this investigation restricted its inclusion to studies published in English and did not search non-English databases, a decision grounded in the superior openness and search reproducibility of English-language databases. Nevertheless, this language restriction may have omitted high-quality research published in other languages, possibly introducing language bias and affecting the comprehensiveness of the evidence.

## 5 Conclusions

Prediction models show promising discriminative ability for predicting progression from prediabetes to T2DM. However, existing evidence is constrained by the limited number of studies, inadequate representativeness of the study populations, incomplete model reporting, and insufficient external validation. Future research should focus on expanding multi-center sample sizes, optimizing model development and validation procedures, and strengthening the assessment of calibration and clinical applicability. Such efforts are necessary to develop robust, interpretable, and generalizable prediction models, thereby providing more reliable decision support for the precision prevention of T2DM.

## Data Availability

The original contributions presented in the study are included in the article/**Supplementary Material**. Further inquiries can be directed to the corresponding author.

## Supplementary material

Supplementary Table 1 Literature search strategy.

Supplementary Table 2 Basic characteristics of eligible studies.
