# Supplementary material for: Prediction models for progression from prediabetes to diabetes: a systematic review and meta-analysis
Source: Front Endocrinol (Lausanne). 2026 Jul 8;17:1888466. doi: 10.3389/fendo.2026.1888466 (PMC13388072; doi:10.3389/fendo.2026.1888466)
Supplement: Supplementary Table 1 — Literature search strategy. [file Table1.docx]

**Table S1 Literature search strategy**

**1. Pubmed**

| Search number | Query | Results |
| --- | --- | --- |
| #1 | "Prediabetic State"[Mesh] | 10404 |
| #2 | "Prediabetic State"[Title/Abstract] OR "Prediabetic States"[Title/Abstract] OR "Prediabetes"[Title/Abstract] OR "Pre-diabetes"[Title/Abstract] OR "pre diabetes"[Title/Abstract] OR "preclinical diabetes"[Title/Abstract] | 13513 |
| #3 | #1 or #2 | 16977 |
| #4 | "machine learning"[MeSH Terms] | 94115 |
| #5 | "machine learning"[Title/Abstract] OR "artificial intelligence"[Title/Abstract] OR "Ensemble Learning"[Title/Abstract] OR "Transfer Learning"[Title/Abstract] OR "prediction model"[Title/Abstract] OR "risk model"[Title/Abstract] OR "risk score"[Title/Abstract] OR "Deep learning"[Title/Abstract] OR "CNN"[Title/Abstract] OR "ResNet"[Title/Abstract] OR "AlexNet"[Title/Abstract] OR "VGGNet"[Title/Abstract] OR "GoogLeNet"[Title/Abstract] OR "random forest"[Title/Abstract] OR "neural network"[Title/Abstract] OR "neural networks"[Title/Abstract] OR "deep networks"[Title/Abstract] OR "deep network"[Title/Abstract] OR "K-Nearest Neighbor"[Title/Abstract] OR "Support vector machine"[Title/Abstract] OR "SVM"[Title/Abstract] OR "Gradient Boosting Machine"[Title/Abstract] OR "Nomogram"[Title/Abstract] OR "XGBoost"[Title/Abstract] OR "Adaboost"[Title/Abstract] OR "LightGBM"[Title/Abstract] OR "Boosting"[Title/Abstract] OR "CatBoost"[Title/Abstract] OR "Gradient Boosting"[Title/Abstract] OR "Decision tree"[Title/Abstract] OR "Regression Trees"[Title/Abstract] OR "Naive Bayesian"[Title/Abstract] OR "Multilayer perceptron"[Title/Abstract] OR "Bayesian network"[Title/Abstract] | 494582 |
| #6 | #4 or #5 | 501310 |
| #7 | #3 and #6 | 520 |

**2.Cochrane**

| Search number | Query | Results |
| --- | --- | --- |
| #1 | MeSH descriptor: [Prediabetic State] explode all trees | 1819 |
| #2 | (“Prediabetic State” OR “Prediabetic States” OR “Prediabetes” OR “Pre-diabetes” OR “pre diabetes” OR “preclinical diabetes”):ti,ab,kw | 4035 |
| #3 | MeSH descriptor: [Machine Learning] explode all trees | 1116 |
| #4 | (“machine learning” OR “artificial intelligence” OR “Ensemble Learning” OR “Transfer Learning” OR “prediction model” OR “risk model” OR “risk score” OR “Deep learning” OR “CNN” OR “ResNet” OR “AlexNet” OR “VGGNet” OR “GoogLeNet” OR “random forest” OR “neural network” OR “neural networks” OR “deep networks” OR “deep network” OR “K-Nearest Neighbor” OR “Support vector machine” OR “SVM” OR “Gradient Boosting Machine” OR “Nomogram” OR “XGBoost” OR “Adaboost” OR “LightGBM” OR “Boosting” OR “CatBoost” OR “Gradient Boosting” OR “Decision tree” OR “Regression Trees” OR “Naive Bayesian” OR “Multilayer perceptron” OR “Bayesian network”):ti,ab,kw | 18221 |
| #5 | #1 or #2 | 4035 |
| #6 | #3 or #4 | 18223 |
| #7 | #5 and #6 | 122 |

**3.Embase**

| Search number | Query | Results |
| --- | --- | --- |
| #1 | 'prediabetic state':ab,ti OR 'prediabetic states':ab,ti OR 'prediabetes':ab,ti OR 'pre-diabetes':ab,ti OR 'pre diabetes':ab,ti OR 'preclinical diabetes':ab,ti | 21386 |
| #2 | 'machine learning'/exp | 592885 |
| #3 | 'machine learning':ab,ti OR 'artificial intelligence':ab,ti OR 'ensemble learning':ab,ti OR 'transfer learning':ab,ti OR 'prediction model':ab,ti OR 'risk model':ab,ti OR 'risk score':ab,ti OR 'deep learning':ab,ti OR 'cnn':ab,ti OR 'resnet':ab,ti OR 'alexnet':ab,ti OR 'vggnet':ab,ti OR 'googlenet':ab,ti OR 'random forest':ab,ti OR 'neural network':ab,ti OR 'neural networks':ab,ti OR 'deep networks':ab,ti OR 'deep network':ab,ti OR 'k-nearest neighbor':ab,ti OR 'support vector machine':ab,ti OR 'svm':ab,ti OR 'gradient boosting machine':ab,ti OR 'nomogram':ab,ti OR 'xgboost':ab,ti OR 'adaboost':ab,ti OR 'lightgbm':ab,ti OR 'boosting':ab,ti OR 'catboost':ab,ti OR 'gradient boosting':ab,ti OR 'decision tree':ab,ti OR 'regression trees':ab,ti OR 'naive bayesian':ab,ti OR 'multilayer perceptron':ab,ti OR 'bayesian network':ab,ti | 574552 |
| #4 | #2 OR #3 | 857261 |
| #5 | #1 AND #4 | 741 |

**4.Web of science**

| Search number | Query | Results |
| --- | --- | --- |
| #1 | “Prediabetic State” OR “Prediabetic States” OR “Prediabetes” OR “Pre-diabetes” OR “pre diabetes” OR “preclinical diabetes” (Topic) | 14373 |
| #2 | “machine learning” OR “artificial intelligence” OR “Ensemble Learning” OR “Transfer Learning” OR “prediction model” OR “risk model” OR “risk score” OR “Deep learning” OR “CNN” OR “ResNet” OR “AlexNet” OR “VGGNet” OR “GoogLeNet” OR “random forest” OR “neural network” OR “neural networks” OR “deep networks” OR “deep network” OR “K-Nearest Neighbor” OR “Support vector machine” OR “SVM” OR “Gradient Boosting Machine” OR “Nomogram” OR “XGBoost” OR “Adaboost” OR “LightGBM” OR “Boosting” OR “CatBoost” OR “Gradient Boosting” OR “Decision tree” OR “Regression Trees” OR “Naive Bayesian” OR “Multilayer perceptron” OR “Bayesian network” (Topic) | 1743334 |
| #3 | #1 AND #2 | 534 |

**Table S2 Basic characteristics of eligible studies**

| No. | First author | Publication year | Country of the first author | Study type | Source of patients | Diagnostic criteria for prediabetes | Follow-up duration | The number of type 2 diabetes cases | Total number of cases (the number of people with prediabetes | Total number of cases in the training set | The generation method of the validation set | The number of cases in the validation set | Model type | Modeling variables |
| --- | --- | --- | --- | --- | --- | --- | --- | --- | --- | --- | --- | --- | --- | --- |
| 1 | Joseph Aoki | 2024 | USA | Cohort study | Registry database | glycosylated hemoglobin 5.0%-6.4% | 5 years | 2129 | 15029 | 12023 | Random sampling | 3006 | RF | Initial A1C, initial serum glucose, A1C slope, serum glucose slope, initial HDL, HDL slope, age, and sex |
| 2 | Arinze Nkemdirim Okere | 2025 | USA | Cohort study | Single center | glycosylated hemoglobin 5.7%-6.4% | 10 years | 426 | 1910 | 1528 | Random sampling | 382 | LR, SVC, KNN, GaussianNB, BernoulliNB, AdaBoost, DT, RF, GB, XGBoost, ET, SHAP | Time points, antidiabetic, dietary counseling, BMI, Black of African American, medicaid, high school graduate, charity/commercial, age, DBP, SBP, gender, CCI, self-pay, exercise frequency, White, less than high school, medicare, other race, above some college, hypothyroidism, some college, statin, HLD, aspirin, diabetes history, Afib, Plavix, hyperthyroidism |
| 3 | Avivit Cahn | 2019 | Israel | Cohort study | Multicenter | glucose ≥ 100 mg/dL and/or HbA1c ≥ 5.7 | 5 years | 160433 | 1217792 | 852454 | Random sampling, external validation | 532717 | Gradient Boosted Trees LightGBM package | 69 selected variables (derived from 11 core signals): age, gender, BMI, glucose, HbA1C, BMI, TG, ALT, WBC, Statins usage, HDL, aspirin usage |
| 4 | Xiaohong Chen | 2024 | China | Cross-sectional study | Single center | 5.6 mmol/L<fasting blood-glucose<7.0 mmol/L or 5.7%<HbA1c<6.5% |  | 616 | 2929 | 2050 | Random sampling | 2929*0.3=879 | LR, RF, SVM, XGBoost | Age, gender, BMI, SBP, U.GLU, PRO, ALT, and TG, AST |
| 5 | Pilar Fuster-Parra | 2023 | Spain | Cohort study | Single center | FPG: 100–125 mg/dl | 5 years | 3,714 | 16648 | 16648 | Cross validation |  | Bayesian networks LR Naïve Bayes Random forest Multilayer perceptron ID3 | Age, gender, educationlevel, and social status, BMI, WC, SPB, DBP, glucose, TG, GGT, and cholesterol, FLI |
| 6 | Di Gong | 2023 | China | Cross-sectional study | Single center | fasting blood-glucose 6.1-6.9 mmol/L, or blood glucose after OGTT 7.8-11.0 mmol/L |  | 567 | 3150 | 2520 | Random sampling | 3150*0.2=630 | Univariable and multivariable logistic regression analysis, full variable model (full.model), simplified model (simp.model) | Glucose metabolism status, age, gender, BMI, SBP, U.GLU, PRO, TP, ALB, GLB, T.BIL, DB, IB, ALT, AST, BUN, SCr, UA, TC, TG, HDL-C, and LDL-C. |
| 7 | Yong Han | 2022 | China | Cohort study | Registry database | 5.6mmol/L<FPG<6.9 mmol/L | 5 years | 2640 | 26018 | 12947 | Random sampling | 13071 | Nomogram of Cox regression | Age, BMI, fasting blood-glucose, TG, ALT, HDL-C, family history of diabetes |
| 8 | Jianshu Yang | 2024 | China | Cohort study | Single center | 6.1≤fasting blood-glucose <7.0 mmol/L and/or 5.7%≤ HbA 1c <6.5% | 3 years | 760 | 4602 | 3221 | Random sampling | 1381 | Multivariable logistic regression analysis, stepwise regression, nomogram | Age, BMI, gender, fasting blood glucose, LDL-C, HDL-C, fatty liver and liver dysfunction |
| 9 | Jiang Li | 2024 | China | Cohort study | Registry database | HbA1c level of 5.7–6.4% (39–47 mmol/mol) | 13.6 years | 2525 | 13489 | 13489*0.8=10791 | Cross validation | 13489*0.2=2698 | Supporting vector machine, RF, and extreme gradient boosting, CPH, RSF | Cholesteryl esters in large HDL, TG in very large VLDL, glycine, average diameter for LDL particles, tyrosine, cholesteryl esters in medium VLDL, glucose, TG in IDL, and docosahexaenoic acid |
| 10 | Kai Liang | 2021 | China | Cohort study | Multicenter | FPG≥5.6 mmol/L and <7.0 mmol/L; 2) , 2hPG ≥7.8 mmol/L and <11.1 mmol/L; 3) HbA1c ≥5.7% and <6.5%. | 3 years | 145 | 1857 |  | Bootstrap internal validation |  |  | Age, gender, BMI, BP, HR, FPG, 2hPG, HbA1c, CR, TC, LDL-c, HDL-c, and TG |
| 11 | Qing Liu | 2022 | China | Cohort study | Single center | FPG≥6.1 mmol/L(110 mg/Dl) and <7.0 mmol/L (126 mg/dL) | 2 years | 3414 | 12009 | 9607 | Random sampling | 2402 | LR, decision tree, RF, and XGBoost | Education, BMI, WC, FPG, TC, TG, HDL-C, and ALT |
| 12 | N. Yokota | 2017 | Japan | Cohort study | Single center | 5.6 mmol/l<FPG<7.0 mmol/l, 7.8mmol/l<2hPG<11.1 mmol/l, 5.7%<HbA1c <6.5% | 4.7 years | 252 | 2105 | 1895 | Cross validation | 210 | RS | Sex, family history of diabetes up to third-degree relatives, SBP, FPG, 1 h-PG (1hPG) and 2hPG during a 75 g OGTT, HbA1c, and ALT |
| 13 | Sia K Nicolaisen | 2022 | Denmark | Cohort study | Registry database | HbA1c: 42-47 mmol/mol (6.0%-6.4%） | Median follow-up time: 2.7 years; maximum duration: 5 years. | 3788 | 26007 | 20806 | Random sampling | 5201 | Fine-Gray competitive risk model + LASSO screening | HbA1c, age, sex, BMI, any antihypertensive drug use, pancreatic disease, cancer, self-­reported diet, doctor’s advice to lose weight or change dietary habits, having someone to talk to, and self-­rated health |
| 14 | Thomas Zueger | 2022 | Switzerland | Cohort study | Registry database | glycosylated hemoglobin 5.7%-6.4% (39-47 mmol/mol) | 5 years | 2102 | 13943 |  | Cross validation |  | SHAP | Age, BMI, glucose, HbA1c, TG, HDL, ALT, and serum creatinine measurements. |
| 15 | Yongsheng Zhang | 2025 | China | Cohort study | Multicenter | HbA 1c5.7%-6.4% | 5 years | 3370 | 8427 | 4,389 | Cross validation | set1:1881 set2: 2157 | LR, RF, SVM, multilayer perceptron, XGBoost, light gradient boosting machine, and categorical boosting machine [CatBoost] | Haematocrit, hemoglobin, RBC, MONO, FBG, HDL/TC, LDL, HDL, creatinine, ALT/AST, age, height, weight, and BMI |
| 16 | Zhuoyang Li | 2025 | China | Cohort study | Single center | h 6.1 ≤ FPG < 7.0 mmol/L or 5.7 ≤ HbA1b < 6.5% | 3.84 years | 374 | 2215 | 1,329 | 10-fold cross validation | 886 | LR, SVM, RF, and XGBoost | Age, income level, FPG, TC, TG, HDL-C, and BMI. |

Abbreviations: Diastolic blood pressure (DBP) systolic blood pressure (SBP), urinary glucose (U.GLU), urinary protein (PRO), total protein (TP), albumin (ALB), globulin (GLB), total bilirubin (T.BIL), direct bilirubin (DB), indirect bilirubin (IB), alanine aminotransferase (ALT), aspartate aminotransferase (AST), blood urea nitrogen (BUN), serum creatinine (SCr), uric acid (UA), total cholesterol (TC), triglycerides (TG), high-density lipoprotein cholesterol (HDL-C), low-density lipoprotein cholesterol (LDL-C), FPG, 1 h-PG (1hPG) ,body mass index (BMI), logistic regression (LR), support vector machine (SVM), random forest (RF), and extreme gradient boosting (XGBoost)
